# Supplementary material for: Link between MHC Fiber Type and Restoration of Dystrophin Expression and Key Components of the DAPC by Tricyclo-DNA-Mediated Exon Skipping
Source: Mol Ther Nucleic Acids. 2017 Oct 26;9:409–18. doi: 10.1016/j.omtn.2017.10.014 (PMC6114118; doi:10.1016/j.omtn.2017.10.014)
Supplement: Document S1. Supplemental Materials and Methods [file mmc1.pdf]

**OMTN, Volume *9***

## Supplemental Information

# Link between MHC Fiber Type and Restoration of Dystrophin Expression and Key Components of the DAPC by Tricyclo-DNA-Mediated Exon Skipping

**Saleh Omairi, Kwan-Leong Hau, Henry Collin-Hooper, Federica Montanaro, Aurelie Goyenvalle, Luis Garcia, and Ketan Patel**

**Supplementary file**

**Primary antibodies for Immunocytochemistry**

| Antigen | Type | Immunoglobulin | Species | Dilution | supplier |
|---|---|---|---|---|---|
| **MYHCIIA** | Monoclonal | IgG | Mouse | 1:1 | DSHB A4.74 |
| **MYHCIIB** | Monoclonal | IgM | Mouse | 1:1 | DSHB BF.F3 |
| **Dystrophin** | Polyclonal | IgG | Rabbit | 1:200 | Abcam, ab15277 |
| **β-SG** | Polyclonal | IgG | Rabbit | 1:200 | Abcam ab203392 |
| **nNOS** | Polyclonal | IgG | Rabbit | 1:200 | Santa cruz Sc-648 |
| **α-DG** | Monoclonal | IgM | mouse | 1:100 | Santa cruz sc-53987 |

**Primary antibodies for Western blotting**

| Antigen | Type | Immunoglobulin | MW | Species | Dilution | supplier |
|---|---|---|---|---|---|---|
| **Dystrophin** | Polyclonal | IgG | 420kDa | Rabbit | 1:200 | Abcam Ab15277 |
| **β-SG** | Polyclonal | IgG | 45 kDa | Rabbit | 1:200 | Abcam ab203392 |
| **nNOS** | Monoclonal | IgG | 160kDa | Mouse | 1:500 | BD Bioscience 610308 |
| **α-DG** | Monoclonal | IgG | 156kDa | Mouse | 1:200 | Millipore 05-593 |
| **GABDH** | Monoclonal | IgG | 38kDa | Mouse | 1-500 | Millipore # MAB374 |

**Secondary antibodies for Immunocytochemistry**

| Antibody | Dilution | Species | supplier |
|---|---|---|---|
| **Alexa fluor 633 anti-mouse** | 1:200 | Goat | Life Technologies # A20146 |
| **Alexa fluor 488 anti-mouse** | 1:200 | Goat | Life Technologies # A11029 |
| **Alexa fluor 488 anti-rabbit** | 1:200 | Goat | Life Technologies # A11034 |
| **Alexa fluor 594 anti-rabbit** | 1:200 | Goat | Life Technologies # A11037 |
