# Supplementary material for: Link between MHC Fiber Type and Restoration of Dystrophin Expression and Key Components of the DAPC by Tricyclo-DNA-Mediated Exon Skipping
Source: Mol Ther Nucleic Acids. 2017 Oct 26;9:409–18. doi: 10.1016/j.omtn.2017.10.014 (PMC6114118; doi:10.1016/j.omtn.2017.10.014)
Supplement: Document S2. Article plus Supplemental Information [file mmc2.pdf]

Molecular Therapy
Nucleic Acids
Original Article

Link between MHC Fiber Type and Restoration of Dystrophin Expression and Key Components of the DAPC by Tricyclo-DNA-Mediated Exon Skipping

Saleh Omairi,[1] Kwan-Leong Hau,[2] Henry Collin-Hooper,[1] Federica Montanaro,[2] Aurelie Goyenvalle,[3] Luis Garcia,[3] and Ketan Patel[1]

[1]School of Biological Sciences, University of Reading, Reading, UK; [2]UCL Great Ormond Street Institute of Child Health, Developmental Neurosciences Programme, London, UK; [3]Universite de Versailles St. Quentin, INSERM U1179, Montigny-le-Bretonneux, France

Exon skipping mediated by tricyclo-DNA (tc-DNA) antisense oligonucleotides has been shown to induce significant levels of dystrophin restoration in *mdx*, a mouse model of Duchenne muscular dystrophy. This translates into significant improvement in key disease indicators in muscle, cardio-respiratory function, heart, and the CNS. Here we examine the relationship between muscle fiber type, based on myosin heavy chain (MHC) profile, and the ability of tc-DNA to restore not only dystrophin but also other members of the dystrophin-associated glycoprotein complex (DAPC). We first profiled this relationship in untreated *mdx* muscle, and we found that all fiber types support reversion events to a dystrophin-positive state, in an unbiased manner. Importantly, we show that only a small fraction of revertant fibers expressed other members of the DAPC. Immunoblot analysis of protein levels, however, revealed robust expression of these components, which failed to correctly localize to the sarcolemma. We then show that tc-DNA treatment leads to nearly all fibers expressing not only dystrophin but also other key components of the DAPC. Of significance, our work shows that MHC fiber type does not bias the expression of any of these important proteins. This work also highlights that the improved muscle physiology following tc-DNA treatment reported previously results from the complete restoration of the dystrophin complex in all MHCII fibers with equal efficiencies.

INTRODUCTION

Duchenne muscular dystrophy (DMD) affects 1:5,000 male births, and it is the most common fatal childhood muscular disease.[1,2] Mutations in the *DMD* gene affect expression of dystrophin, a protein normally localized to the inner surface of the sarcolemma in muscle fibers.[3,4] Dystrophin together with a number of other proteins that constitute the dystrophin-associated glycoprotein complex (DAPC) acts to link the muscle fiber cytoskeleton, the sarcolemma, and the extracellular matrix (ECM) into a functional unit that maintains muscle integrity.[5,6] The DAPC is composed of three sub-complexes: (1) the sarcoglycans (α, β, γ, and δ); (2) syntrophin, nNOS, and dystrobrevin; and (3) α and β dystroglycan. The absence of dystrophin results in a drastic reduction of all components of the DAPC at the sarcolemma, and it renders muscle cells prone to stretch-induced muscle damage.[7]

A number of drug-based or surgical procedures have been developed, including the use of corticosteroids or addressing scoliosis, that greatly improve the quality of life for DMD patients or delay disease onset.[8,9] However, none has completely halted progression of the disease. Gene-based approaches that aim to restore dystrophin in muscle hold great promise. One attractive approach is to take advantage of the molecular structure of the dystrophin gene and to use antisense oligonucleotides (AONs) to promote exon skipping to bypass mutated stretches of DNA and restore the open reading frame.[10] The aim of the current study is to establish expression of a functional, albeit internally deleted, dystrophin protein. Restoration of dystrophin expression by exon skipping has been proven to be efficacious *in vitro*, in animal models and in DMD patients.[11–13] Several classes of chemical modifications have been developed for AON-mediated exon skipping, among which are 2′O-methylribooligonucleoside-phosphorothioate (2′OMe), phosphorodiamidate morpholino oligomers (PMOs), and tricyclo-DNA (tc-DNA). The latter has a number of properties that make it an attractive chemistry to exploit for therapeutic uses, including high RNA affinity, resistance to nuclease activity, and the ability to form nanoparticles that may facilitate uptake into cells.[14–16] We have recently shown, using *mdx* mice as a rodent model for DMD, that tc-DNA mediates unprecedented levels of exon skipping after systemic delivery not only in skeletal muscle but also in the heart and brain.[16] This translated into normalization of specific force in the tibialis anterior muscle as well as improved cardiovascular function and the correction of behavioral characteristics.[16]

A number of studies using AONs in both *mdx* mice and DMD patients have shown restoration of dystrophin in a subset of muscle fibers.[13,17,18] Most skeletal muscles are composed of a heterogeneous

Received 20 July 2017; accepted 21 October 2017;
https://doi.org/10.1016/j.omtn.2017.10.014.

Correspondence: Ketan Patel, School of Biological Sciences, University of Reading, Whiteknights, Reading RG6 6UB, UK.
E-mail: ketan.patel@reading.acc.uk

This is an open access article under the CC BY license (http://creativecommons.org/licenses/by/4.0/).

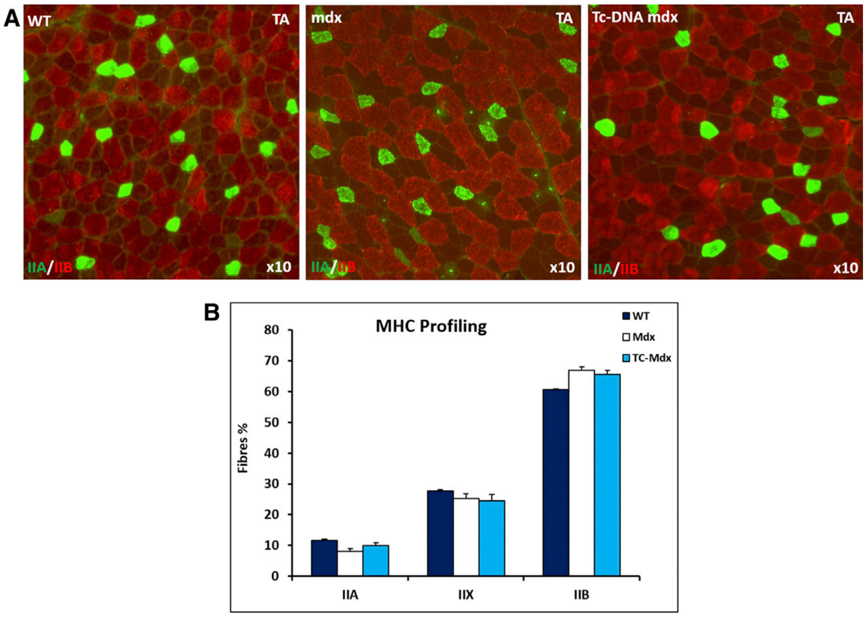

Figure 1. Myosin Heavy Chain Profile of the Tibialis Anterior Muscle

(A) Immunohistochemical images of TA muscle from 20- to 22-week-old male wild-type, *mdx*, and tc-DNA-treated *mdx* mice. Green fibers signify the expression of MHCIIA with MHCIIB appearing as red. Non-green and red fibers represent MHCIIX. (B) MHC profile in the three cohorts. Results show that WT, *mdx*, and tc-DNA-treated *mdx* mice have the same proportion of each MHC subtype (n = 4 for each cohort). Statistical analysis was performed by one-way ANOVA followed by Bonferroni correction for multiple comparison.

population of muscle fibers that differ in their metabolic properties as well as contractile speeds, a feature impacted by the type of myosin heavy chain (MHC) being expressed. Muscle of adult mice is composed of MHCI, MHCIIA, MHCIIX, and MHCIIB fibers. MHCI has the slowest contraction rate and is highly reliant on oxidative phosphorylation for energy production. MHCIIB is at the other end of the spectrum, displaying the fastest contraction rates and highly dependent on glycolytic metabolism. Slow fibers are invested with a higher capillary density as well as thicker ECM compared to fast fibers.[19,20] Fast-contracting fiber with its decreased ability to store energy in the ECM is hypothesized to facilitate a greater proportion of force transfer to the skeletal elements.[21] A number of studies have shown that slow muscle, based on MHC expression profiling as well as physical measures, expresses more dystrophin than fast muscle[22] and that fast muscle fibers are preferentially affected in DMD.[23]

Here we examined whether the efficacy of dystrophin exon skipping is influenced by MHC fiber type, possibly due to fiber type differences in ECM thickness impacting on the rate of AON diffusion into the muscle fiber. We first profiled revertant fibers in *mdx* mice with a view of establishing whether their appearance was related to MHC fiber type. We then investigated the relationship between the restoration of dystrophin and of members of the three DAPC sub-complexes by treatment with tc-DNA and by MHC fiber type. Our results demonstrate that revertant fibers caused by splicing events in untreated *mdx* mice develop in a manner independent of MHC fiber type. Importantly, we show that only a fraction of revertant fibers also express DAPC members. Treatment with tc-DNA results in over 90% of all fibers expressing all proteins examined, with no observed bias toward any one MHC fiber type. These data demonstrate that tc-DNA treatment is able to induce exon skipping in all MHCII fibers.

RESULTS

We first established the MHC landscape of the tibialis anterior (TA) muscle and the effect wrought upon it first by the *mdx* mutation and second after treatment with AONs consisting of tc-DNA. Previous work has reported that the *mdx* mutation affects the MHC fiber profile in a muscle-specific manner, with the extensor digitorum longus (EDL) and soleus unchanged by the mutation[24] whereas the diaphragm contains slower isoforms compared to control.[25] Analysis of the TA muscle of wild-type (WT) mice at its maximum circumference revealed an approximate ratio of 1:3:6 with respect to MHCIIA, MHCIIX, and MHCIIB fibers (Figures 1A and 1B). The same ratios were found in the TA muscles of *mdx* mice and tc-DNA-treated *mdx* mice (Figures 1A and 1B). Statistical analysis failed to reveal significant differences in the proportions of a particular MHC isoform among the three cohorts. Therefore, the MHCII profile of TA muscle was not affected by the absence of dystrophin or by treatment with tc-DNA.

We next examined the relationship between revertant fibers (dystrophin^{+}), co-expression of one member of each of the three DAPC sub-complexes, and MHCII class in untreated *mdx* mice. There were approximately 60 dystrophin^{+} fibers in the TA muscle of 20- to 22-week-old mice. The ratio of dystrophin^{+} in relation to MHC fiber type (IIA:IIX:IIB) was approximately 1:3:6, respectively. Therefore, the segregation of dystrophin^{+} fibers within MHC subtypes followed the distribution of each isoform. Hence, there was no bias toward any one MHC isoform with regard to reversion to a dystrophin-positive state (Figure 2A). Profiling the expression of β-sarcoglycan, nNOS, and α-dystroglycan revealed a number of interesting features. First, they were found in all three MHC fiber isoforms, and, similar to dystrophin, there was no bias toward any one MHCII type (Figure 2A). β-sarcoglycan-, nNOS-, and α-dystroglycan-expressing fibers were a subset of those that expressed dystrophin. However, the number of fibers that expressed these three molecules was always lower than the number expressing dystrophin (Figure 2B). Indeed, nNOS-positive fibers, although being the most frequent of the three, only represented about half of dystrophin-positive fibers.

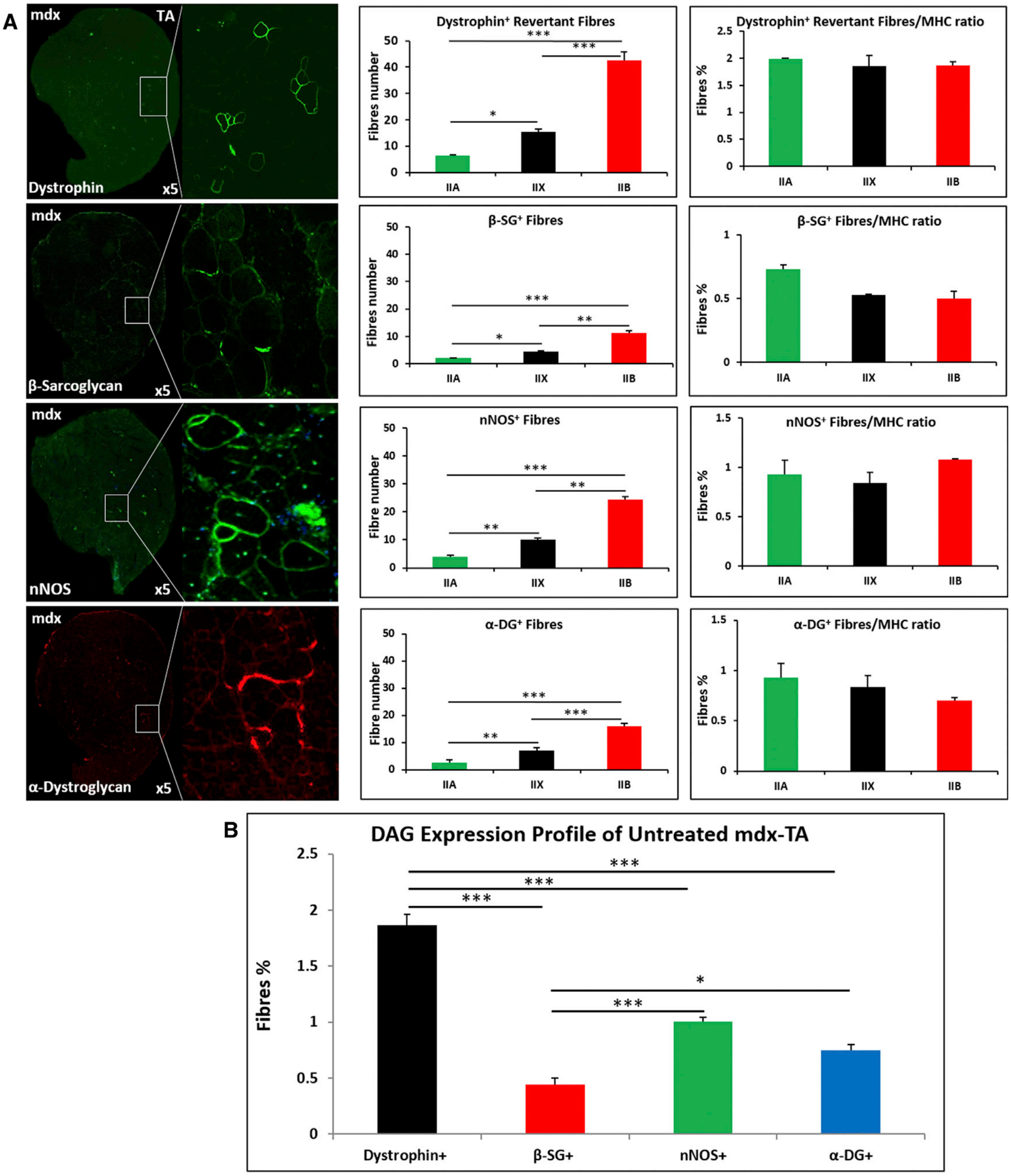

Figure 2. Expression of Dystrophin, β-Sacroglycan, nNOS, and α-Dystroglycan in the TA Muscle of 20- to 22-Week-Old Male *mdx* Mice

(A) Each row shows the entire TA muscle stained for one of the four molecules together with a magnified detailed image. All positive fibers were correlated to the expression of an MHC isoform, and their distribution is given as the total number as well as proportion to the frequency of the MHC isoform. (B) Graph showing the proportion of TA fibers expressing the four investigated molecules (n = 4 for each cohort). *p < 0.05, **p < 0.01, and ***p < 0.001. Statistical analysis was performed by one-way ANOVA followed by Bonferroni correction for multiple comparison.

These results show that revertant fibers do not express the full complement of DAPC components.

Thereafter, we examined the expression of dystrophin, β-sarcoglycan, nNOS, and α-dystroglycan in relation to MHC fiber type in the TA muscle of tc-DNA-treated *mdx* mice. Immunostaining revealed that the majority of fibers were positive for dystrophin after tc-DNA treatment, which is in agreement with previous findings of Goyenvalle and colleagues[16] (Figure 3A). Robust expression of dystrophin was found in all MHC fiber types, and analysis of

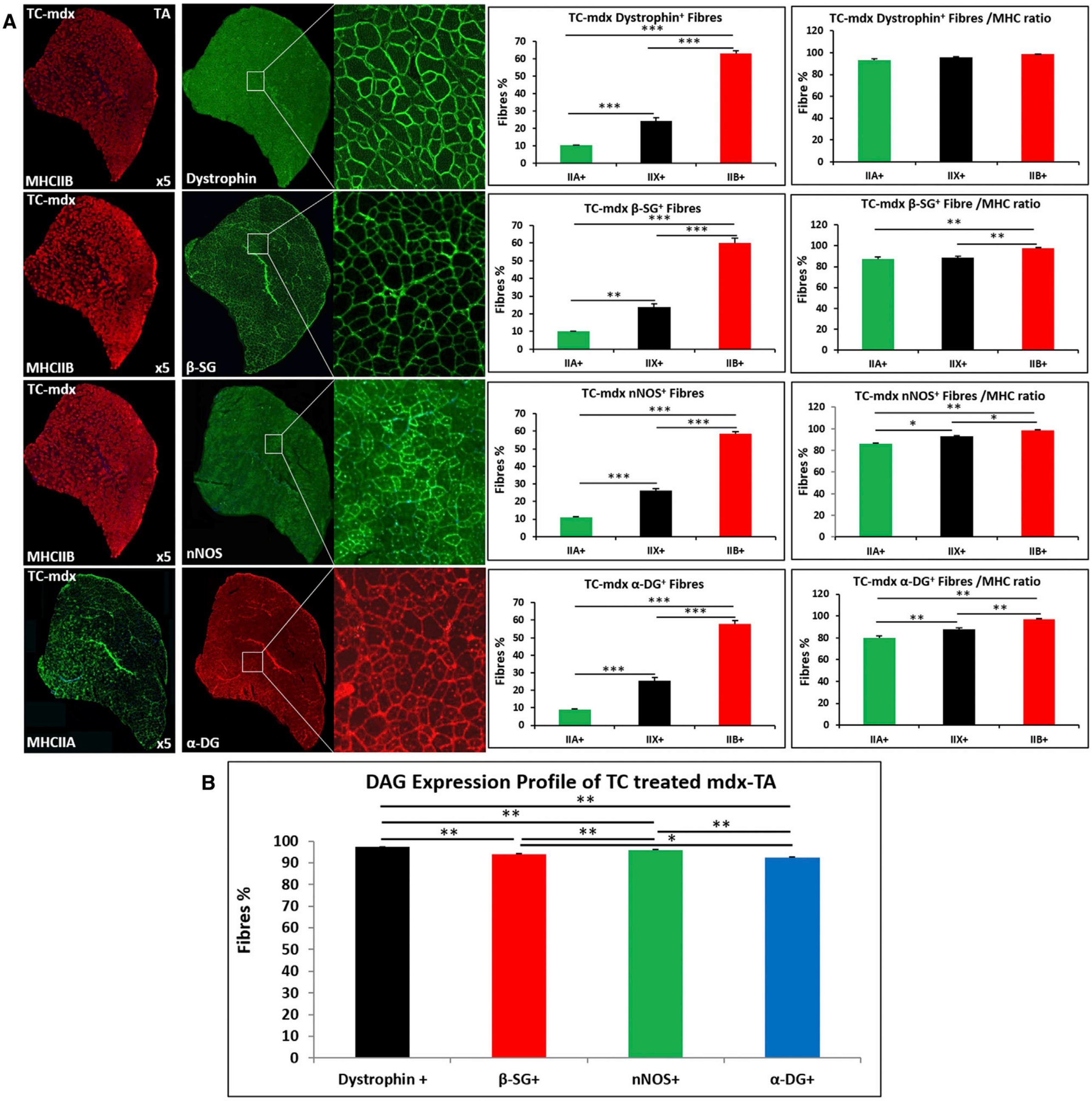

Figure 3. Expression of Dystrophin, β-Sarcoglycan, nNOS, and α-Dystroglycan in the TA Muscle of 20- to 22-Week-Old Male tc-DNA-Treated *mdx* Mice

(A) Each row shows the entire TA muscle immunostained for dystrophin, β-sarcoglycan, nNOS, and α-dystroglycan together with a magnified detailed image. All positive fibers were correlated to the expression of an MHC isoform, and their distribution is given as the total number as well as proportion to the frequency of the MHC isoform. (B) Graph showing the proportion of TA fibers from tc-DNA-treated *mdx* mice expressing the four investigated molecules (n = 4 for each cohort). *p < 0.05, **p < 0.01, and ***p < 0.001. Statistical analysis was performed by one-way ANOVA followed by Bonferroni correction for multiple comparison.

frequency with respect to fiber proportion revealed that there was no bias to any one fiber type. Immunostaining for β-sacroglycan, nNOS, and α-dystroglycan revealed the same features as dystrophin; the vast majority of fibers expressed the three proteins, and their presence in a particular MHC fiber type was proportional to the frequency of that form (Figure 3A). We then compared the relative frequency of fibers expressing each of the four proteins. We found there were significantly more fibers that expressed dystrophin than the other three components of the DAPC assessed (Figure 3B).

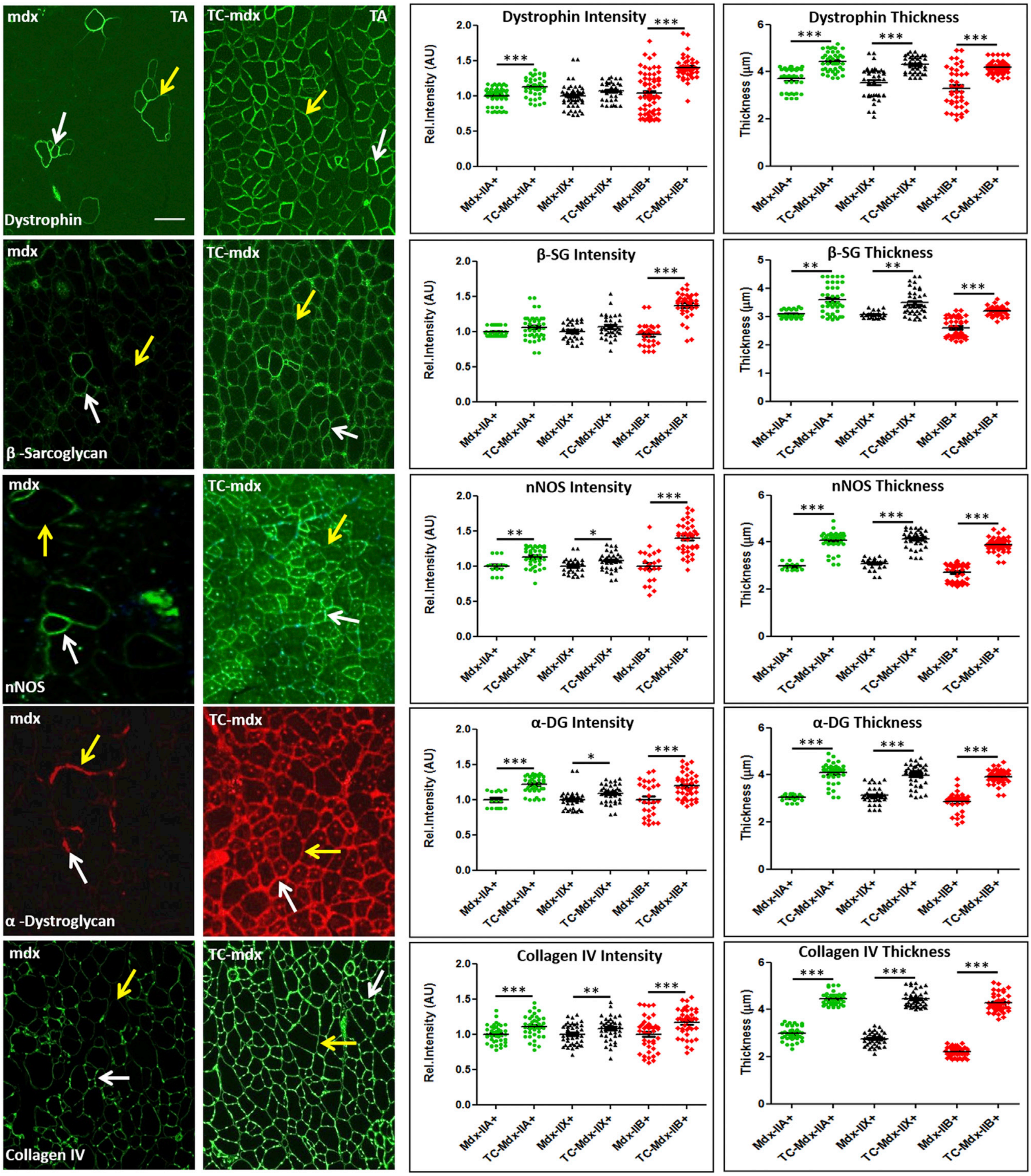

Figure 4. Semiquantitative Analysis of DAPC Restoration following tc-DNA Treatment of 20- to 22-Week-Old Male *mdx* Mice

Untreated and tc-DNA-treated muscle shows high levels of individual proteins in MHCIIA fibers (white arrows) compared to MHCIIB fibers (yellow arrows). Intensities from over 30 regions for each fiber type were taken from revertant untreated *mdx* and set to a reference value of one. Similar numbers of intensity readings were plotted for each fiber

(legend continued on next page)

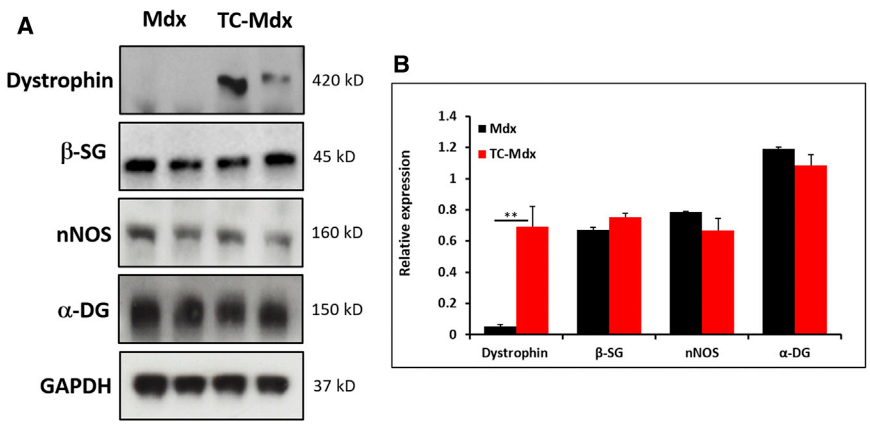

Figure 5. Western Blot Analysis of DAPC Proteins in 20- to 22-Week-Old Male Mice

(A) Western blot image of DAPC proteins in the TA muscle. (B) Quantification of DAPC proteins relative to GAPDH. Note the robust expression of β-sarcoglycan, nNOS, and α-dystroglycan in untreated *mdx* muscle (n = 3 for each cohort). **p < 0.01. Statistical analysis was performed using two-tailed t test.

These results show that tc-DNA treatment of *mdx* mice results in the restoration of dystrophin in the majority of muscle fibers. This is also the case for α-dystroglycan, β-sarcoglycan, and nNOS. However, the number of fibers expressing β-sarcoglycan, nNOS, and α-dystroglycan was significantly lower compared to dystrophin.

Previous work has shown that contractile properties of a muscle fiber impact both qualitatively and quantitatively on its surrounding ECM.[19,20] Here we examined the relationship between MHC fiber type and expression of components of the DAPC as well as an ECM component, collagen IV, using semiquantitative techniques. Fluorescence intensity was used as previously described to gain an indication of the amount of protein at the sarcolemma.[18] We first determined the signal intensity for the five proteins in question in relation to MHC fiber type in the revertant fibers from the *mdx* mouse. For each fiber type, the signal intensity was set to a reference value of 1. Thereafter the same procedure was repeated for the tc-DNA-treated muscle, and intensity was compared to that of the untreated TA muscle. The outcome of the process showed that tc-DNA treatment resulted in an increase in the amount of each protein of interest in all fiber types compared to untreated revertant *mdx* fibers (Figure 4). We also measured the thickness of the expression domain for each of the five marker proteins, revealing that each expression domain was thicker in MHCIIA fibers compared to MHCIIB fibers (Figure 4). This relationship persisted following tc-DNA treatment. Second, we found that there was an increase in the expression domain following tc-DNA treatment for all fiber types (Figure 4).

These results show that the amount of each component of the DAPC and collagen IV at the sarcolemma were elevated above those found in revertant fibers.

Last, we examined the effect of tc-DNA treatment on the total level of expression of the DAPC components under investigation here. To that end we carried out quantitation of western blots. Our results showed that there was a 14-fold increase in the amount of dystrophin following tc-DNA treatment (Figures 5A and 5B). Interestingly, we found robust expression of β-sarcoglycan, nNOS, and α-dystroglycan in untreated *mdx* muscle and that their levels were not changed significantly by tc-DNA treatment. In summary, components of the DAPC are translated in the absence of dystrophin, but they fail to localize to the sarcolemma.

DISCUSSION

Tc-DNA chemistry is an exciting development in the area of molecular medicine. We have previously shown that, in the context of a mouse model of DMD, tc-DNA treatment was more efficacious in restoring muscle function than many other approaches.[16] Of particular note was the finding that tc-DNA AONs spontaneously form nanoparticles, which are believed to promote entry into the cell and may be the reason why these were able to penetrate the heart and the brain.[16] In this study, we investigated the relationship between muscle compositions in terms of MHC fiber type and dystrophin restoration by tc-DNA AON with a view to developing an understanding of its specificity of action.

We commenced the study by comparing the MHC profile of the TA muscle in the three cohorts under investigation: WT, *mdx*, and tc-DNA-treated *mdx* mice. We found that all three shared the same MHC profile. Previous studies have shown that the MHC profile of the diaphragm underwent a significant change in its MHC compositions, with a decrease in the proportion of MHCIIB$^+$ fibers and a concomitant increase in the number of MHCI$^+$ fibers.[25] The change in MHC profile was proposed to be an adaptive step to preserve contractile function and fiber integrity by lowering energy requirements. In contrast, the EDL and soleus muscles of the *mdx* were shown to be identical to those from WT in terms of MHC profile.[24] Our results here now add the TA muscle to the list of muscles that show normal MHC profile in the *mdx* mouse. Nevertheless, all *mdx* muscles have reduced specific force. We propose that, if a change toward a slower MHC profile is an adaptive change to the absence of dystrophin, it must be a secondary step that is dependent on other upstream factors,

type from tc-DNA-treated *mdx* muscle. In all cases there was a significant increase in intensity compared to untreated revertant fibers of the same MHC type. Thickness of expression domain was measured and plotted for each MHC isoform originating from revertant untreated *mdx* and tc-DNA-treated *mdx* muscle. MHCIIA fibers had thicker expression domains compared to MHCIIB. Thickness for expression domains irrespective of MHC fiber type was increased by tc-DNA treatment (n = 4 for each cohort). Scale bar applicable to all images represents 50 μm. *p < 0.05, **p < 0.01, and ***p < 0.001. Statistical analysis was performed using two-tailed t test.

one of which could be contractile activity, which would explain the change in constantly used muscles like the diaphragm and not in limb muscles. A number of studies have proposed mechanisms to explain the reduced specific force in *mdx*, including nitrosylation of the contractile machinery, which, if it occurred, would lead to long-term damage due to low turnover rate of MHC.[26,27] However, recent work has shown that the MHC from *mdx* functions normally in terms of cross-bridging, which argues against long-term effect of altered muscle function.[28] This suggestion is indirectly corroborated by the efficacy of tc-DNA treatment being able to normalize specific force in the TA muscle of *mdx* mice.[16]

Our work examining the distribution of the DAPC protein in untreated *mdx* muscle offers interesting insights into the formation of the functional unit. First, we show that there was no bias in terms to MHC fiber type and the appearance of dystrophin. Therefore, if metabolic activity were to generate differential cellular stress based on fiber contraction rate, then this metric does not impact on the splicing events that restore dystrophin expression in *mdx* muscle. Furthermore, when serendipitous events lead to the restoration of dystrophin, they bring back the protein in a relatively normal manner with respect to MHC fiber type, with higher expression in slow fibers compared to faster ones.[22] Additionally, there was no bias in terms of MHC fiber type and any of the other components of the DAPC examined here.

An interesting feature highlighted by our work in this section was the finding that revertant fibers (dystrophin$^+$) are heterogeneous in terms of their DAPC composition as follows: dystrophin$^+$ > nNOS$^+$ > α-dystroglycan (αDG)$^+$ > β-sarcoglycan (ßSG)$^+$. Our quantifications of DAPC expression in revertant fibers extends previous findings of Lu et al.[29] who showed co-expression of DAPC proteins in clusters of revertant fibers. Interestingly, our western blotting data agree with prior reports showing an abundance of nNOS, αDG, and ßSG total protein in *mdx* muscle.[30] It follows, therefore, that a mechanism must be active that prevents the translocation of DAPC proteins to the sarcolemma of some revertant fibers. One possibility is that they may not have had sufficient time to correctly translocate. This is, however, unlikely since revertant fibers form from events that occur in muscle precursors.[31] Another possibility is linked to the poor diffusion of dystrophin within the myofiber sarcolemma, limiting membrane expression to spatially confined nuclear domains. Moving out of this dystrophin domain during serial sectioning would affect detection of other components of the DAPC. While this phenomenon can contribute to a decreased co-detection of dystrophin and DAPC proteins, it should be noted that dystrophin expression in revertant fibers has been reported to span membrane segments of 654 ± 409 μm.[32] It is unlikely that exiting a nuclear domain during collection of serial sections over a length of muscle not exceeding 160 μm (16 serial sections) could on its own account for over half of the revertant fibers lacking expression of other DAPC proteins (Figure 3B). It is therefore possible that a significant proportion of internally deleted dystrophins generated by revertant fibers is not able to assemble a functional DAPC but can be correctly localized to the membrane. This hypothesis would be consistent with reports of truncated or internally deleted dystrophins that lack the cysteine-rich domain required for interaction with the DAPC but can still be correctly localized to the sarcolemma.[33] Overall our results highlight two interesting findings: first, the majority of revertant fibers produce internally deleted forms of dystrophin that cannot functionally contribute to force transduction, since they are uncoupled from the dystroglycan and sarcoglycan complexes; and, second, *mdx* muscle has a rich pool of DAPC proteins available for recruitment to the sarcolemma upon expression of a functional dystrophin protein.

Restoration of dystrophin expression following tc-DNA treatment resulted in near total coverage of TA fibers, consistent with our previous work.[16] We do not believe that the variation in the affinities of antibodies for their epitopes is a decisive factor in showing a variation in DAPC profile between *mdx* and tc-*mdx* mice. We base this conclusion on the fact that have we have compared the same strain (indeed littermates) with or without tc-DNA treatment. Therefore, the differing affinities between antibodies for their particular epitope would remain a constant factor. Hence, the appearance of a molecule at the sarcolemma in tc-*mdx* mice compared to *mdx* must be due to changes in the expression levels of the protein. The results of this study demonstrate that there is no bias with regard to dystrophin expression induction following tc-DNA treatment and fiber type. This is, we believe, highly relevant and important for prospective translation into therapies.

Previous work carried out in humans revealed restoration of dystrophin in a subset of muscle fibers, a differential restoration that may have been influenced by the structural properties of the muscle.[13,17] Indeed, it is well established that slow muscle fibers have a thicker ECM in comparison to fast fibers.[19,20] It would, therefore, be reasonable to postulate that slow fibers are more resistant to infiltration by tc-DNA AON. However, our work shows that, at least in terms of type II fiber sub-types, there is no preference to exon skipping. This bodes well for the use of this chemistry in a spectrum of muscles with differing fiber composition, as it seems they are all in principle able to take up the tc-DNA AON. In addition, we show that MHC fiber type does not influence the restoration of the other components examined here. Also, tc-DNA treatment leads to more of each component at the sarcolemma compared to revertant *mdx* fibers. Nevertheless, it is worth noting that not all the fibers that expressed dystrophin contained the other three components of the DAPC examine here (dystrophin$^+$ > nNOS$^+$ > ßSG$^+$ > αDG$^+$). This again highlights the point that the presence of the DAPC proteins does not necessarily translate into them being assembled into a functional complex.

There is a dearth of knowledge regarding mechanisms that regulate the formation of the DAPC, a gap in our understanding that requires urgent attention. One potential consequence of this gap in our understanding is that it is possible that we will develop the means of inducing protein dystrophin expression but that it may not

translocate to the correct sub-cellular region and, therefore, reduce therapeutic benefit. Insights into this process could be gained by examining revertant fibers. Nevertheless, tc-DNA treatment resulted in over 90% of the fibers having all four of the DAPC components at the sarcoplasm. We believe that this high level of DAPC restoration explains the normalization of specific force following tc-DNA treatment, and, again, it bodes well for translation into the clinic since previous studies have demonstrated that restoration of dystrophin protein levels to 10%–20% of WT results in improved health.[34]

In summary, we show that reversion of fibers to a dystrophin-positive state in *mdx* mice is a stochastic process with regard to MHC fiber type. However, expression of dystrophin in *mdx* revertant fibers only translates into a minority (>25%) of fibers expressing members of the three sub-complexes. Tc-DNA treatment results in over 90% of fibers' expression of dystrophin as well as members of the three sub-complexes in the TA muscle. Importantly, there is no bias in terms of expression of any component with regard to MHC fiber type. This work shows that, in principle, tc-DNA treatment is equally efficacious across all type II fibers.

MATERIALS AND METHODS

Animals

Animal procedures were performed in accordance with national and European legislation, approved by the French government (Ministère de l'enseignement supérieur et de la recherche, Autorisation APAFiS 6518). *Mdx* (C57BL/10ScSc-Dmdmdx/J) and C57BL/10 mice were bred in our specific opportunistic pathogen-free (SOPF) animal facility at the Plateform 2Care, UFR des Sciences de la santé, Université de Versailles Saint Quentin, and they were maintained in a standard 12-hour light/dark cycle with free access to deionized water and standard laboratory chow (M20, Dietex) *ad libitum*. Mice were weaned at postnatal weeks 4–5 and 2–5 individuals were housed per cage. Mice were randomly allocated to treatment and control groups, ensuring equal numbers of control and treated mice within the same litters.

The tc-DNA-AON PS M23D (5′-AACCTCGGCTTACCT-3′) targeting the donor splice site of exon 23 of the mouse dystrophin pre-mRNA used in this study was synthesized by SYNTHENA (Bern, Switzerland). The 6- to 8-week-old male *mdx* mice were injected intravenously in the retro-orbital sinus, under general anesthesia using 1.5%–2% isoflurane, once a week with 200 mg/kg/week of the M23D-tc-DNA for a period of 12 weeks. Treated mice were sacrificed 2 weeks after the last injection, and muscles and tissues were harvested and snap-frozen in liquid nitrogen-cooled isopentane and stored at −80°C before further analysis.

Immunohistochemistry

Dissected and frozen muscles were mounted in Tissue Tech freezing medium (Jung) cooled by dry ice/ethanol. Immunohistochemistry staining was performed on 10-μm cryosections that were dried for 30 min at room temperature (RT) prior to three washes in 1× PBS. Muscle sections were incubated in permeabilization buffer solution (0.952 g HEPES, 0.260 g $MgCl_2$, 0.584 g NaCl, 0.1 g Sodium azide, 20.54 g Sucrose, and 1 mL Triton X-100) for 15 min at room temperature, before the application of block wash buffer (PBS with 5% fetal calf serum [v/v] and 0.05% Triton X-100) for 30 min at room temperature.

Primary antibodies were pre-blocked in wash buffer for 30 min prior to incubation onto muscle sections overnight at 4°C. Pre-blocked-in wash buffer was performed for all secondary antibodies (in dark) for a minimum of 30 min prior to their addition onto the slides. Sections were then incubated for 1 hr in the dark at room temperature. Finally, slides were mounted in fluorescent mounting medium, and myonuclei were visualized using 2.5μg/mL DAPI. Details of primary and secondary antibodies are given in Supplemental Materials and Methods.

Western Blotting

TA proteins from 20- to 22-week-old male mice (20 μg/lane) were separated on 4%–12% gradient SDS-PAGE gels (Invitrogen), transferred to nitrocellulose membranes (Whatman), and blocked with 5% skim milk in 0.1% Tween 20/Tris-buffered saline. Membranes were cut at appropriate molecular weights in order to allow for simultaneous probing of the exact same samples for dystrophin and multiple DAPC proteins. Membrane strips were then incubated with appropriate primary antibodies overnight at 4°C, followed by a 1-hr incubation at room temperature with the appropriate horseradish peroxidase-conjugated secondary antibodies (Jackson ImmunoResearch Laboratories). Protein bands were visualized using enhanced chemiluminescence reagents (Pierce). Signal was detected on X-ray film (RPI) at multiple exposures. For densitometric analysis, protein band intensities from multiple non-saturated film exposures were quantified using ImageJ (NIH). Values in the linear range of pixel intensities were selected for quantifications. Signal intensities were normalized to GAPDH, used as an internal loading control, and probed on the same membrane. Details of primary and secondary antibodies are given in Supplemental Materials and Methods.

Semiquantitative Measures of Sarcolemma Protein Expression

Intensity of signals of protein of interest was measured as previously described.[18] Briefly, membrane signal intensities of approximately 30 muscle fibers of each MHC phenotype (IIA, IIX, and IIB) in each TA muscle section from *mdx* mice and *mdx* mice treated with tc-DNA were measured. Fiji software was used to measure signal from area of interest after images had been corrected for background to avoid regions of signal saturation. To calculate relative signal intensity levels, individual measurements from treated and control fibers were taken as a percentage of mean of control samples.

Sarcolemma Thickness Measurement

Connective tissue thickness between approximately 30 fibers of the same MHC phenotypes (IIA-IIA, IIX-IIX, and IIB-IIB) of TA muscle sections was measured using Fiji software. One measurement on the constant connective tissue thickness and multiple measurements on the fluctuating connective tissue thickness areas between each two myofibers that expressed the same MHC isoform were taken on all muscle sections of *mdx* mice and *mdx* mice treated with tc-DNA.

Imaging and Analysis

A fluorescence microscope (Zeiss AxioImegar A1) was used to examine immunofluorescently stained sections, and images were captured using an Axiocam digital camera with Zeiss Axiovision computer software version 4.8.

Statistical Analysis

Data are presented as mean ± SE. Significant differences between two groups were performed by two-tailed Student's t test for independent variables. Differences among groups were analyzed by one-way ANOVA followed by Bonferroni multiple comparison tests as appropriate. Statistical analysis was performed on GraphPad Prism software. Differences were considered statistically significant at $^{*}p < 0.05$, $^{**}p < 0.01$, or $^{***}p < 0.001$.

SUPPLEMENTAL INFORMATION

Supplemental Information includes Supplemental Materials and Methods and can be found with this article online at https://doi.org/10.1016/j.omtn.2017.10.014.

AUTHOR CONTRIBUTIONS

Conceptualization, K.P.; Methodology, A.G. and L.G.; Investigation, S.O., K.-L.H., H.C.-H., and F.M.; Writing, S.O., F.M., A.G., L.G., and K.P.; Supervision, K.P.

ACKNOWLEDGMENTS

This work was supported by the Ministry of Higher Education of Iraq, the Agence Nationale de la Recherche (France), the Association Monegasque Contre les Myopathies (Monaco), and the Biotechnology and Biological Sciences Research Council (BBSRC-UK) (J016454/1 to H.C.-H.). F.M. is supported by a European Horizon 2020 re-integration Marie-Skłodowska Curie Senior Fellowship. We are also grateful to two anonymous reviewers for comments that have greatly improved the manuscript. Finally, we are indebted to Dr. Patrick Lewis for proofreading the revised version.

REFERENCES

1. Hoffman, E.P., and Connor, E.M. (2013). Orphan drug development in muscular dystrophy: update on two large clinical trials of dystrophin rescue therapies. Discov. Med. *16*, 233–239.

2. Chung, J., Smith, A.L., Hughes, S.C., Niizawa, G., Abdel-Hamid, H.Z., Naylor, E.W., Hughes, T., and Clemens, P.R. (2016). Twenty-year follow-up of newborn screening for patients with muscular dystrophy. Muscle Nerve *53*, 570–578.

3. Zubrzycka-Gaarn, E.E., Bulman, D.E., Karpati, G., Burghes, A.H., Belfall, B., Klamut, H.J., Talbot, J., Hodges, R.S., Ray, P.N., and Worton, R.G. (1988). The Duchenne muscular dystrophy gene product is localized in sarcolemma of human skeletal muscle. Nature *333*, 466–469.

4. Hoffman, E.P., Brown, R.H., Jr., and Kunkel, L.M. (1987). Dystrophin: the protein product of the Duchenne muscular dystrophy locus. Cell *51*, 919–928.

5. Ohlendieck, K., Ervasti, J.M., Snook, J.B., and Campbell, K.P. (1991). Dystrophin-glycoprotein complex is highly enriched in isolated skeletal muscle sarcolemma. J. Cell Biol. *112*, 135–148.

6. Dickson, G., Azad, A., Morris, G.E., Simon, H., Noursadeghi, M., and Walsh, F.S. (1992). Co-localization and molecular association of dystrophin with laminin at the surface of mouse and human myotubes. J. Cell Sci. *103*, 1223–1233.

7. Matsumura, K., Tomé, F.M., Ionasescu, V., Ervasti, J.M., Anderson, R.D., Romero, N.B., Simon, D., Récan, D., Kaplan, J.C., Fardeau, M., et al. (1993). Deficiency of dystrophin-associated proteins in Duchenne muscular dystrophy patients lacking COOH-terminal domains of dystrophin. J. Clin. Invest. *92*, 866–871.

8. Kim, S., Campbell, K.A., Fox, D.J., Matthews, D.J., and Valdez, R.; MD STARnet (2015). Corticosteroid Treatments in Males With Duchenne Muscular Dystrophy: Treatment Duration and Time to Loss of Ambulation. J. Child Neurol. *30*, 1275–1280.

9. Lee, J.W., Won, Y.H., Choi, W.A., Lee, S.K., and Kang, S.W. (2013). Successful surgery for scoliosis supported by pulmonary rehabilitation in a duchenne muscular dystrophy patient with forced vital capacity below 10%. Ann. Rehabil. Med. *37*, 875–878.

10. Wilton, S.D., and Fletcher, S. (2008). Exon skipping and Duchenne muscular dystrophy: hope, hype and how feasible? Neurol. India *56*, 254–262.

11. Alter, J., Lou, F., Rabinowitz, A., Yin, H., Rosenfeld, J., Wilton, S.D., Partridge, T.A., and Lu, Q.L. (2006). Systemic delivery of morpholino oligonucleotide restores dystrophin expression bodywide and improves dystrophic pathology. Nat. Med. *12*, 175–177.

12. Arechavala-Gomeza, V., Graham, I.R., Popplewell, L.J., Adams, A.M., Aartsma-Rus, A., Kinali, M., Morgan, J.E., van Deutekom, J.C., Wilton, S.D., Dickson, G., and Muntoni, F. (2007). Comparative analysis of antisense oligonucleotide sequences for targeted skipping of exon 51 during dystrophin pre-mRNA splicing in human muscle. Hum. Gene Ther. *18*, 798–810.

13. Kinali, M., Arechavala-Gomeza, V., Feng, L., Cirak, S., Hunt, D., Adkin, C., Guglieri, M., Ashton, E., Abbs, S., Nihoyannopoulos, P., et al. (2009). Local restoration of dystrophin expression with the morpholino oligomer AVI-4658 in Duchenne muscular dystrophy: a single-blind, placebo-controlled, dose-escalation, proof-of-concept study. Lancet Neurol. *8*, 918–928.

14. Renneberg, D., Bouliong, E., Reber, U., Schümperli, D., and Leumann, C.J. (2002). Antisense properties of tricyclo-DNA. Nucleic Acids Res. *30*, 2751–2757.

15. Murray, S., Ittig, D., Koller, E., Berdeja, A., Chappell, A., Prakash, T.P., Norrbom, M., Swayze, E.E., Leumann, C.J., and Seth, P.P. (2012). TricycloDNA-modified oligo-2′-deoxyribonucleotides reduce scavenger receptor B1 mRNA in hepatic and extra-hepatic tissues–a comparative study of oligonucleotide length, design and chemistry. Nucleic Acids Res. *40*, 6135–6143.

16. Goyenvalle, A., Griffith, G., Babbs, A., El Andaloussi, S., Ezzat, K., Avril, A., Dugovic, B., Chaussenot, R., Ferry, A., Voit, T., et al. (2015). Functional correction in mouse models of muscular dystrophy using exon-skipping tricyclo-DNA oligomers. Nat. Med. *21*, 270–275.

17. van Deutekom, J.C., Janson, A.A., Ginjaar, I.B., Frankhuizen, W.S., Aartsma-Rus, A., Bremmer-Bout, M., den Dunnen, J.T., Koop, K., van der Kooi, A.J., Goemans, N.M., et al. (2007). Local dystrophin restoration with antisense oligonucleotide PRO051. N. Engl. J. Med. *357*, 2677–2686.

18. Cirak, S., Feng, L., Anthony, K., Arechavala-Gomeza, V., Torelli, S., Sewry, C., Morgan, J.E., and Muntoni, F. (2012). Restoration of the dystrophin-associated glycoprotein complex after exon skipping therapy in Duchenne muscular dystrophy. Mol. Ther. *20*, 462–467.

19. Elashry, M.I., Collins-Hooper, H., Vaiyapuri, S., and Patel, K. (2012). Characterisation of connective tissue from the hypertrophic skeletal muscle of myostatin null mice. J. Anat. *220*, 603–611.

20. Kovanen, V., Suominen, H., and Heikkinen, E. (1980). Connective tissue of "fast" and "slow" skeletal muscle in rats–effects of endurance training. Acta Physiol. Scand. *108*, 173–180.

21. Kovanen, V., Suominen, H., and Heikkinen, E. (1984). Mechanical properties of fast and slow skeletal muscle with special reference to collagen and endurance training. J. Biomech. *17*, 725–735.

22. Ho-Kim, M.A., and Rogers, P.A. (1992). Quantitative analysis of dystrophin in fast- and slow-twitch mammalian skeletal muscle. FEBS Lett. *304*, 187–191.

23. Webster, C., Silberstein, L., Hays, A.P., and Blau, H.M. (1988). Fast muscle fibers are preferentially affected in Duchenne muscular dystrophy. Cell *52*, 503–513.

24. Anderson, J.E., Bressler, B.H., and Ovalle, W.K. (1988). Functional regeneration in the hindlimb skeletal muscle of the mdx mouse. J. Muscle Res. Cell Motil. *9*, 499–515.

25. Petrof, B.J., Stedman, H.H., Shrager, J.B., Eby, J., Sweeney, H.L., and Kelly, A.M. (1993). Adaptations in myosin heavy chain expression and contractile function in dystrophic mouse diaphragm. Am. J. Physiol. *265*, C834–C841.

26. Li, D., Yue, Y., Lai, Y., Hakim, C.H., and Duan, D. (2011). Nitrosative stress elicited by nNOSμ delocalization inhibits muscle force in dystrophin-null mice. J. Pathol. *223*, 88–98.

27. Guellich, A., Negroni, E., Decostre, V., Demoule, A., and Coirault, C. (2014). Altered cross-bridge properties in skeletal muscle dystrophies. Front. Physiol. *5*, 393.

28. Bates, G., Sigurdardottir, S., Kachmar, L., Zitouni, N.B., Benedetti, A., Petrof, B.J., Rassier, D., and Lauzon, A.M. (2013). Molecular, cellular, and muscle strip mechanics of the mdx mouse diaphragm. Am. J. Physiol. Cell Physiol. *304*, C873–880.

29. Lu, Q.L., Morris, G.E., Wilton, S.D., Ly, T., Artem'yeva, O.V., Strong, P., and Partridge, T.A. (2000). Massive idiosyncratic exon skipping corrects the nonsense mutation in dystrophic mouse muscle and produces functional revertant fibers by clonal expansion. J. Cell Biol. *148*, 985–996.

30. Rezniczek, G.A., Konieczny, P., Nikolic, B., Reipert, S., Schneller, D., Abrahamsberg, C., Davies, K.E., Winder, S.J., and Wiche, G. (2007). Plectin 1f scaffolding at the sarcolemma of dystrophic (*mdx*) muscle fibers through multiple interactions with β-dystroglycan. J. Cell Biol. *176*, 965–977.

31. Yokota, T., Lu, Q.L., Morgan, J.E., Davies, K.E., Fisher, R., Takeda, S., and Partridge, T.A. (2006). Expansion of revertant fibers in dystrophic mdx muscles reflects activity of muscle precursor cells and serves as an index of muscle regeneration. J. Cell Sci. *119*, 2679–2687.

32. Chretien, F., Dreyfus, P.A., Christov, C., Caramelle, P., Lagrange, J.L., Chazaud, B., and Gherardi, R.K. (2005). In vivo fusion of circulating fluorescent cells with dystrophin-deficient myofibers results in extensive sarcoplasmic fluorescence expression but limited dystrophin sarcolemmal expression. Am. J. Pathol. *166*, 1741–1748.

33. Rafael, J.A., Cox, G.A., Corrado, K., Jung, D., Campbell, K.P., and Chamberlain, J.S. (1996). Forced expression of dystrophin deletion constructs reveals structure-function correlations. J. Cell Biol. *134*, 93–102.

34. Hoffman, E.P., Bronson, A., Levin, A.A., Takeda, S., Yokota, T., Baudy, A.R., and Connor, E.M. (2011). Restoring dystrophin expression in duchenne muscular dystrophy muscle progress in exon skipping and stop codon read through. Am. J. Pathol. *179*, 12–22.

OMTN, Volume *9*

Supplemental Information

Link between MHC Fiber Type and Restoration of Dystrophin Expression and Key Components of the DAPC by Tricyclo-DNA-Mediated Exon Skipping

Saleh Omairi, Kwan-Leong Hau, Henry Collin-Hooper, Federica Montanaro, Aurelie Goyenvalle, Luis Garcia, and Ketan Patel

Supplementary file

Primary antibodies for Immunocytochemistry

Antigen	Type	Immunoglobulin	Species	Dilution	supplier
**MYHCIIA**	Monoclonal	IgG	Mouse	1:1	DSHB A4.74
**MYHCIIB**	Monoclonal	IgM	Mouse	1:1	DSHB BF.F3
**Dystrophin**	Polyclonal	IgG	Rabbit	1:200	Abcam, ab15277
**β-SG**	Polyclonal	IgG	Rabbit	1:200	Abcam ab203392
**nNOS**	Polyclonal	IgG	Rabbit	1:200	Santa cruz Sc-648
**α-DG**	Monoclonal	IgM	mouse	1:100	Santa cruz sc-53987

Primary antibodies for Western blotting

Antigen	Type	Immunoglobulin	MW	Species	Dilution	supplier
**Dystrophin**	Polyclonal	IgG	420kDa	Rabbit	1:200	Abcam Ab15277
**β-SG**	Polyclonal	IgG	45 kDa	Rabbit	1:200	Abcam ab203392
**nNOS**	Monoclonal	IgG	160kDa	Mouse	1:500	BD Bioscience 610308
**α-DG**	Monoclonal	IgG	156kDa	Mouse	1:200	Millipore 05-593
**GABDH**	Monoclonal	IgG	38kDa	Mouse	1-500	Millipore # MAB374

Secondary antibodies for Immunocytochemistry

Antibody	Dilution	Species	supplier
**Alexa fluor 633 anti-mouse**	1:200	Goat	Life Technologies # A20146
**Alexa fluor 488 anti-mouse**	1:200	Goat	Life Technologies # A11029
**Alexa fluor 488 anti-rabbit**	1:200	Goat	Life Technologies # A11034
**Alexa fluor 594 anti-rabbit**	1:200	Goat	Life Technologies # A11037
